# Molecular Mechanisms of Drug-Induced Hemolysis in G6PD Deficiency: Mechanistic Insights

**DOI:** 10.1155/omcl/7041213

**Published:** 2025-08-05

**Authors:** Sulaiman Paika, Matthew Machini, Mayur S. Parmar

**Affiliations:** Dr. Kiran C. Patel College of Osteopathic Medicine, Nova Southeastern University, Clearwater, Florida, USA

## Abstract

Glucose-6-phosphate dehydrogenase (G6PD) deficiency, a prevalent enzymopathy, predisposes individuals to hemolytic anemia upon exposure to various medications. This literature review explores the molecular underpinnings of drug-induced hemolytic anemia (DIHA) in G6PD-deficient patients, focusing on dapsone, amoxicillin, and primaquine. These drugs are essential for treating infections such as leprosy and malaria. However, they can damage red blood cell (RBC) membranes through complex mechanisms distinct from traditional immune-mediated pathways. Evidence suggests that drug metabolites, such as dapsone hydroxylamine and 5-hydroxyprimaquine, induce oxidative stress and disrupt RBC membrane integrity. The band 3 protein, a critical component of the RBC cytoskeleton, emerges as a key player in this process, undergoing tyrosine phosphorylation and aggregation, leading to membrane remodeling and instability. This review underscores the need for further research to elucidate the precise molecular interactions involved in drug-induced hemolysis in G6PD deficiency. Understanding these mechanisms may pave the way for developing targeted therapies, including adjuvant treatments and novel drug formulations, to mitigate the risk of hemolytic anemia in this vulnerable population.

## 1. Introduction

Glucose-6-phosphate dehydrogenase (G6PD) deficiency is characterized by a lack of the G6PD enzyme ubiquitously in red blood cells (RBCs). G6PD plays a highly critical role in preventing cellular damage from reactive oxidative species, thus facilitating membrane stability along with other critical molecular components [1]. Deficiency in such an enzyme leads to a marked decrease in membrane integrity, which increases susceptibility to oxidative damage when utilizing pharmacological agents, ultimately resulting in hemolytic events.

G6PD is one of the most common enzyme deficiencies affecting nearly 400 million individuals worldwide. Still, a higher prevalence of the mutation is apparent in tropical regions, often associated with areas where malaria is endemic. Evidence shows that mutations resulting in G6PD deficiency and sickling may have been introduced as a genetic advantage against plasmodium infections [2]. G6PD deficiency has a genetic etiology introduced by inherited X-linked aberrant mutations in the *G6PD* gene, leading to reduced activity or stability of the corresponding enzyme. Over 200 identifiable mutations are associated with the *G6PD* gene, making it common in both U.S. and global populations. The severity of symptoms differs across populations, but in a general sense, most individuals with G6PD deficiency are relatively asymptomatic. However, a threat is posed when those individuals are introduced to several pharmacological agents, resulting in hemolysis [3].

G6PD's catalytic role in the pentose phosphate pathway (PPP) facilitates the reduction of NADP to NADPH, a vital protective molecule against oxidative damage. When NADPH is deficient, there is no catalytic reaction to activate glutathione reductase, which converts hydrogen peroxide to water, thereby, preventing reactive oxidative species from threatening the integrity of RBC membranes. Offending agents, further exacerbating this process of RBC membrane degeneration and cell death, cause intrinsic hemolytic anemia in which there is a substantial decrease in hemoglobin due to erythrocyte death [4].

Various documented agents have been shown to precipitate hemolytic anemia in patients with G6PD deficiency. The known medication classes such as antimalarial drugs, sulfonamide drugs, aspirin, and other antimicrobial agents have been implicated in drug-induced hemolysis following administration, making the utilization of these agents clinically challenging for patients with G6PD deficiency [5]. While there is a general understanding of how these drugs may induce hemolytic anemia through oxidative damage, a standardized mechanism still lacks in which metabolites of certain medications may interact with critical components of the RBC membrane.

Current accepted mechanisms of drug-induced hemolytic anemia (DIHA) point to an immunological etiology. Drug-induced immune hemolytic anemia (DIIHA) can be characterized as a type II hypersensitivity reaction in which drug-dependent or independent antibodies are formed against either the drug–RBC fusion complex or against RBCs, directly killing erythrocytes, resulting in intravascular hemolysis [6]. The former mechanism places forth an idea that upon metabolism, the drug may coat the RBC and IgG antibodies to form a response against receptors of the drug, which has been supported through various research studies as well as clinical serum testing [6]. However, the underlying mechanism of RBC membrane damage is still poorly understood. Furthermore, novel research shows that some medications have been shown to affect the erythrocyte membrane without immunological absorption taking place, heavily contesting previous notions of immune-mediated pathways [6].

Regardless of whether damage to RBC membrane integrity is initiated immunologically or via direct chemical effects, further research is crucial to elucidate the underlying molecular mechanisms of membrane instability. This research should focus specifically on how drug metabolites interact with critical RBC membrane components like the band 3 protein, the nature of subsequent alterations to the cytoskeleton, and the role of oxidative stress in initiating protein aggregation and signaling pathway disruptions. Recent findings also emphasize the metabolic impact of drug metabolites, such as DDS-NHOH's disruption of glycolysis in G6PD-deficient RBCs, highlighting a need to explore both membrane and metabolic vulnerabilities [7]. Once discovered, appropriate adjustments to pharmacological structure, or perhaps novel adjuvant therapies to decrease hemolytic stress, may be created. This is especially important in populations endemic with both malaria and G6PD deficiency, as the medications used to treat them have been implicated in inducing hemolytic anemia.

This literature review aims to examine and assess research about novel mechanisms in DIHA, with a particular focus on dapsone, amoxicillin, and primaquine. These drugs were chosen due to their significant risk of triggering hemolysis in G6PD-deficient individuals, their diverse range of potential mechanisms for inducing hemolysis (including both drug metabolite-induced oxidative stress and potential non-immune mediated membrane damage), and their clinical relevance in regions where G6PD deficiency and the diseases they treat (malaria and leprosy) are prevalent. By providing insights into these specific drugs, we hope to contribute to developing strategies to better manage and prevent hemolytic anemia in this vulnerable population.

## 2. Specific DIHA in G6PD-Deficient Individuals

**Figure 1:**
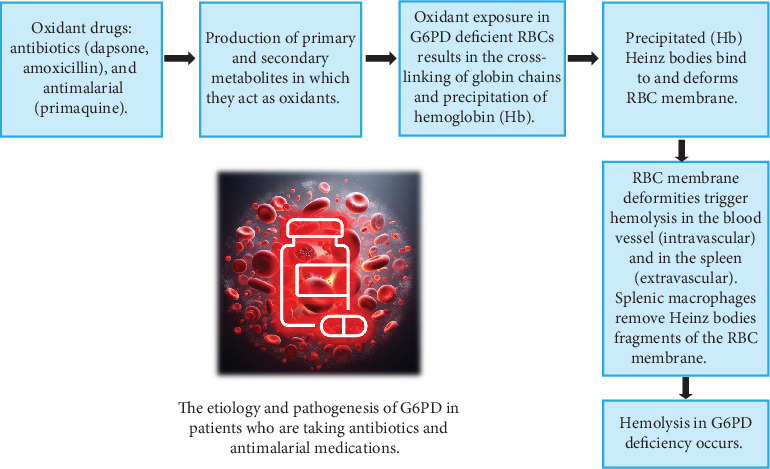
The illustration of one of the molecular mechanisms underlying drug-induced hemolytic anemia in G6PD-deficient individuals. The diagram illustrates the role of oxidant drugs, such as dapsone, amoxicillin, and primaquine, in generating reactive oxygen species (ROS). These ROS led to the cross-linking and precipitation of hemoglobin (Hb) into Heinz bodies, which bind to the red blood cell (RBC) membrane, causing deformation, and subsequent hemolysis.

Recognizing the coexistence of both traditional immune responses and direct non-immune effects in drug-induced hemolysis is crucial for understanding drug toxicity, especially in vulnerable G6PD-deficient populations.  Table 1 summarizes the key distinguishing features, including mediators, molecular targets, and diagnostic indicators associated with each pathway, providing a framework for the specific drug examples detailed later. A key non-immune pathway common to many oxidant drugs, involving the formation of Heinz bodies from damaged hemoglobin, is outlined in Figure 1.

### 2.1. Dapsone

Dapsone, an aniline derivative, is categorized as a sulfa drug utilized in a broad spectrum against commensals. However, its proprietary use is against mycobacterium, specifically *M. leprae*. Additionally, dapsone has been utilized in conjunction with pyrimethamine to combat malaria. Like sulfonamides, dapsone exerts its pharmacological activity by inhibiting folic acid synthesis, specifically by inhibiting the synthesis of dihydrofolic acid by competition with para-aminobenzoic acid (PAB) at the dihydropteroate synthetase active site [8].

#### 2.1.1. Metabolism and Pharmacokinetics

Pharmacologically, dapsone is absorbed rapidly in all tissues with a roughly 70%–80% bioavailability postoral administration, with a renal elimination half-life of approximately 28 h [9]. One of the main side effects of dapsone, in relevance to this paper, is the induction of hemolytic anemia, which is especially evident in G6PD-deficient patients. Dapsone has pharmacogenomic adverse reactions with over 150 G6PD alleles, resulting in hemolytic anemia. While the causes of DIHA are multifarious and largely point towards an immune-mediated response, there is still much left to be elucidated regarding the actual underlying mechanism [6]. Furthermore, the mechanism of action in agents that cause hemolytic anemia may vary depending on the drug. However, one aspect remains unchanged: The centralized destruction of the RBC wall integrity. The mechanisms by which dapsone engages in the destruction of RBC walls have been discussed in the relevant literature.

More recent discoveries point towards a specific dapsone metabolite, dapsone-N-hydroxylamine, as the perpetrator. Crucially, studies have confirmed that direct exposure of erythrocytes to the parent dapsone drug fails to trigger membrane protein phosphorylation. In contrast, the hydroxylamine metabolite (DDS-NHOH) readily initiates the damaging cascade (dapsone hydroxylamine induces premature removal of human erythrocytes by membrane reorganization and antibody binding). Regarding its metabolic activity, cytochrome P450 2E1 (CYP2E1)-mediated hepatic metabolism of dapsone results in the immediate formation of dapsone hydroxylamine and nitroso-dapsone, which have also been implicated in hemolytic anemia pathogenesis [10]. It was found that individuals with G6PD have a twofold sensitivity towards dapsone-induced hemolytic anemia, which is mediated by N-hydroxy-dapsone metabolites; however, the mechanism is unknown [11]. Exposure to the dapsone metabolites was shown to cause premature splenic sequestration of damaged RBCs. Recent in vivo studies using a humanized mouse model (hG6PDMed-) have confirmed that DDS-NHOH selectively causes clearance of G6PD-deficient RBCs, with lower doses leading to temporary sequestration and higher doses (e.g., 1000 μM) resulting in permanent removal, unlike G6PD-sufficient RBCs, which mostly return to circulation [7]. It can be said that dapsone and its subsequent metabolite-mediated RBC wall damage are dose-dependent, as another study showed that the concentration required to induce erythrocyte damage is much higher in human red cells than in rat RBCs [12].

#### 2.1.2. RBC Membrane Effects

A mechanism proposed by McMillan et al. [13] stipulated that the hemolytic activity of the dapsone metabolite (DDS-NOH) results in disulfide-linked hemoglobin accumulation on membrane skeletal proteins and that the loss of structural integrity is derived from the induction of oxidative stress, further resulting in premature splenic sequestration of RBCs. Furthermore, it was theorized that increased oxidative stress could lead to lipid peroxidation of the RBC wall layer. However, this was disproved as the data supporting such actions was insignificant [13]. The generation of reactive oxidative species is a highly contended mechanism in dapsone-mediated hemolytic anemia. DDS-NHOH has been shown to convert hemoglobin to methemoglobin in greater amounts, which may increase reactive oxygen species (ROS). In G6PD-deficient patients, there is an existing defect in ROS detoxification via GSH, resulting in decreased RBC integrity, making it far easier for dapsone metabolites to enact damage [13]. This concept is vividly demonstrated in populations with elevated baseline oxidative stress, such as individuals with endometriosis. Research indicates that their erythrocytes display a markedly increased susceptibility to DDS-NHOH, showing greater band 3 phosphorylation and aggregation than those of control subjects. This highlights how an existing pro-oxidant condition significantly exacerbates dapsone-related toxicity [14]. However, dapsone may induce hemolytic anemia independently of G6PD status, but there is a greater risk of RBC destruction in the presence of G6PD deficiency.

There is definite merit in looking at dapsone's implication in the induction of hemolytic anemia, excluding patients with G6PD deficiency, as it may allow for further exploration into the underlying pharmacological mechanism. Understanding how dapsone alone, without compounding variables, can damage an erythrocyte's architectural integrity may benefit pharmaceutical development. According to a series of case studies reported by Lee and Geetha [15] involving patients with renal dysfunction, higher doses of dapsone correlated with increased hemolysis. However, all four patients were placed on immunosuppressant therapies. Tacrolimus competes with dapsone for P-450 metabolism, which may have increased the probability of hemolytic induction. Regardless, it was shown that without pre-existing damage to RBC wall integrity by G6PD deficiency, dapsone is pharmacologically able to induce hemolysis in a dose-dependent fashion, indicating that there must be an independent mechanism arbitrating RBC membrane destruction [15].

#### 2.1.3. RBC Cytoarchitecture and Integrity

Before discussing novel pathways involved in dapsone-mediated hemolytic anemia, one must understand the RBC cytoarchitecture regarding integrity and ion exchange. RBC's membrane skeleton is inherently adaptive for its physiological role, composed of a lipid bilayer with complex scaffolding networks weaved by hexagonal F-actin alignment. While most cells are spheroid in shape, erythrocytes adopt a biconcave shape to allow for greater surface area for oxygen loading and unloading, implying that membrane permeability is highly important. This is accomplished by formations of junctional complexes in parallel order, creating a quasi-hexagonal lattice that is composed mainly of a protein named band 3 [16]. In addition to oxygen permeability, RBCs must contain a membrane structure conducive to ion exchange to maintain adequate acid–base balance. Vertical linkages between the cytoskeleton and membrane allow for the creation of such channels, which are maintained by the support of the band 3 protein [16].

#### 2.1.4. Role of Band 3

**Figure 2:**
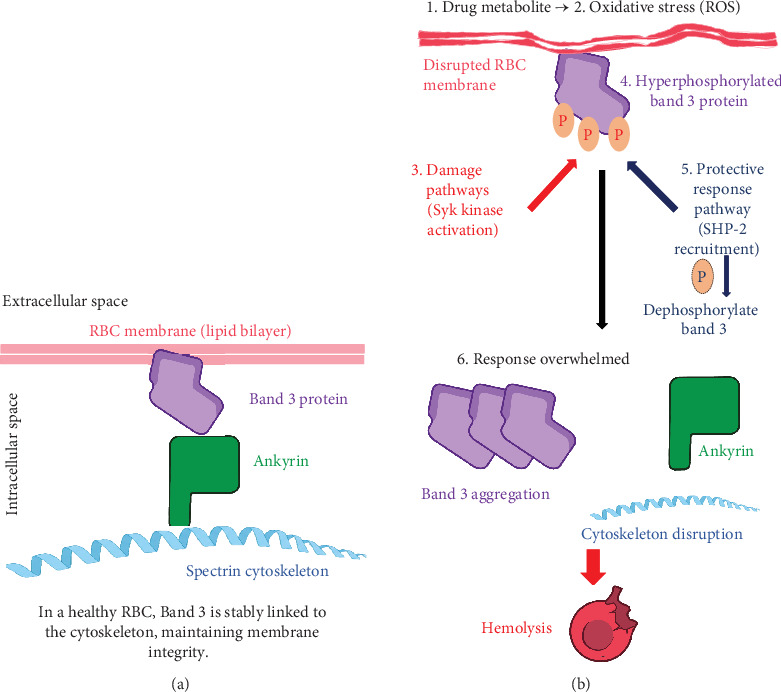
The proposed Interaction of Syk and SHP-2 with band 3 in drug-induced hemolysis. This diagram illustrates the “damage and response” cascade leading to red blood cell (RBC) membrane instability, contrasting the stable state of a normal RBC with the pathological events initiated by oxidant drug metabolites. Panel (A) normal red blood cell. This panel illustrates a healthy erythrocyte where the band 3 protein is securely anchored to the underlying spectrin cytoskeleton via the ankyrin protein. This stable structural linkage is crucial for maintaining membrane integrity. Panel (B) drug-induced damage. This panel illustrates the step-by-step pathological process: (1) drug metabolite: an active drug metabolite (e.g., dapsone hydroxylamine and DDS-NHOH) is introduced, leading to (2) oxidative stress: the generation of reactive oxygen species (ROS) and a state of oxidative stress, which causes (3) Syk kinase activation: the activation of the protein tyrosine kinase Syk, the primary initiator of damage, results in (4) band 3 hyperphosphorylation: the addition of multiple phosphate groups to the band 3 protein. In response, the cell initiates a (5) compensatory SHP-2 recruitment: a protective response where the protein tyrosine phosphatase SHP-2 is recruited to counteract the damage by attempting to dephosphorylate band 3. Ultimately, this leads to (6) response overwhelmed and hemolysis: a state where the protective SHP-2 response is insufficient, causing a net accumulation of hyperphosphorylated, aggregated band 3. This results in cytoskeletal disruption, irreversible membrane instability, and hemolysis.

As the most abundant membrane protein, band 3 maintains mechanical support by linking itself to the ankyrin protein and engaging in bicarbonate transport activity. Interestingly, dysfunction in the band 3 protein has been implicated in RBC wall destruction in both G6PD deficiencies and dapsone-induced hemolysis [17]. As mentioned, the dapsone metabolite DDS-NHOH has been implicated in RBC wall damage through unknown mechanisms. Still, novel research may show a connection between said metabolites and detrimental tyrosine phosphorylation of band 3 protein, leading to ubiquitous membrane remodeling [10]. With the variable of G6PD deficiency added, there is already a proposed increase in membrane remodeling along with ROS-mediated damage, causing widespread membrane integrity loss, so adding toxic dapsone metabolites would surely result in further damage. It has been shown that there are higher levels of band 3 accumulation in G6PD patients, resulting in membrane remodeling [17]. However, with the addition of dapsone metabolites, hemolytic anemia may ensue. It is possible that, through damaging oxidative pathways, DDS-NHOH can further progress the aggregation of band 3 protein, leading to autoantibody destruction via autologous antibody recognition, ultimately disrupting ion exchange channels and subsequent RBC wall integrity [18]. In a more molecular sense, the interaction of DDS-NHOH with the erythrocyte membrane initiates a dynamic “damage and response” cascade centered on band 3. Research has shown this process involves the simultaneous recruitment of two opposing enzymes to the membrane: the protein tyrosine kinase Syk, which phosphorylates band 3 and drives the damage, and the protein tyrosine phosphatase SHP-2, which is recruited to dephosphorylate band 3 as a compensatory, protective response [17]. Recent metabolic analyses further reveal that DDS-NHOH induces a blockage in terminal glycolysis in G6PD-deficient RBCs, leading to pyruvate accumulation due to inhibited lactate dehydrogenase (LDH) activity, exacerbated by impaired PPP flux and NADPH depletion, compounding membrane damage [7]. The presence and relocation of SHP-2 can, therefore, be viewed as a molecular marker of the membrane's altered state. In cases of severe or prolonged exposure to the dapsone metabolite, this protective phosphatase system is ultimately overwhelmed, leading to a net increase in phosphorylated and aggregated band 3, cytoskeletal disruption, and hemolysis [17]. This proposed molecular cascade, initiated by the drug metabolite and mediated by SHP-2, is illustrated in Figure 2.

Evidence highlights the complex interplay between dapsone metabolites, oxidative stress, and band 3 protein dysfunction in triggering hemolysis in G6PD-deficient individuals. However, other than dapsone, amoxicillin, a widely used antibiotic, has also been implicated in hemolytic anemia, even in cases without G6PD deficiency.

### 2.2. Amoxicillin

Amoxicillin, created in 1972, is one of the most ubiquitously utilized pharmacological agents within the beta-lactam class of antibiotics. Derived semi-synthetically from penicillin, with an additional amino group, amoxicillin is frequently prescribed to cover broad-spectrum gram-positive and some gram-negative bacteria. FDA-approved indications include many infections, ranging from tonsillitis to *H. pylori* eradication. Off-label usage encompasses treatment for infectious endocarditis, Lyme disease, and actinomycoses [19]. The amoxicillin mechanism of action falls under the paradigm in which beta-lactams operate by inhibiting the binding of bacterial penicillin proteins (PBP-1), thereby, eliminating the crosslinking of D-alanine and D-aspartic acid, causing damage to bacterial cell walls [19]. However, amoxicillin is often given in concert with a beta-lactamase inhibitor such as clavulanic acid [19].

#### 2.2.1. Pharmacokinetics and Safety

Regarding pharmacodynamics, amoxicillin has a bioavailability of 60%, a half-life of 61.3 min, and is metabolized by the liver into seven identifiable metabolites [20]. Amoxicillin is relatively safe but may cause a variety of side effects ranging from nausea to hypersensitivity vasculitis. Mainly, amoxicillin's adverse effects are relegated to allergic and hypersensitivity reactions [19]. However, several case studies have noted hemolytic anemia with and without G6PD deficiency, but the mechanism of action remains a question.

#### 2.2.2. Mechanisms of Hemolytic Anemia

It is important to note that it is often challenging to distinguish DIIHA from other causes [21]. Failure to arrive at the correct primary cause of a complication, such as hemolytic anemia, can be detrimental, leading to increased mortality. The mechanism of DIIHA has been studied extensively, with recent research providing various antibody-mediated mechanisms of action. However, a potential novel mechanism has been identified specifically in antibiotics non-immune protein adsorption (NIPA), proposing that properties of certain drugs, such as beta-lactamase inhibitors, can modify erythrocyte membrane integrity through various mechanisms, namely destructive oxidation reactions [6].

#### 2.2.3. Immune-Mediated Hemolysis

A case study published by Chan Gomez et al. [21] demonstrated DIHA 5 days post-amoxicillin–clavulanate prescription for a sinus infection. Clinical presentation on admission included dark urine and jaundice, with no signs of hepatosplenomegaly. Confirmation of DIIHA using direct antiglobulin test (DAT) showed elevated levels of C3 bound to RBC membranes, indicating drug-independent autoantibody formation [21]. However, the mechanism of RBC membrane damage from autoantibody binding is unknown.

#### 2.2.4. Non-Immune-Mediated Hemolysis and G6PD/G6PI Deficiency

While many cases of DIHA involve antibody/immune-mediated RBC membrane destruction, a case of amoxicillin-induced hemolytic anemia in a child with glucose 6-phosphate isomerase deficiency proposed a non-immune-mediated paradigm [22]. Certain clinical aspects of this case argue against DIIHA etiology, such as the absence of anti-reticulocyte antibodies, indicating a lack of an immune-mediated response. Another study by Blanquicett et al. [23] noticed reduced reticulocyte count and negative Coombs' test, providing further evidential support against immune-mediated RBC membrane destruction. It has been shown that G6PI and G6PD deficiencies induce a vulnerable state in RBC architecture, making it less difficult for damaging agents to affect the membrane integrity [5].

#### 2.2.5. Therapeutic Implications and Future Directions

While oxidative damage has been implicated in membrane loss of integrity, it does not explain the acuity in which the damage occurs, nor how reactive oxidative species degenerate membrane structure. Researchers propose that novel insights into G6PD polymorphisms will facilitate the elucidation of susceptibility in G6PD-deficient patients. However, it may be more prudent to understand the underlying molecular mechanism by which agents such as amoxicillin enact RBC membrane damage exclusively from pathologies affecting erythrocyte stability. Future studies on the integral molecular components involved in RBC membrane destruction are necessary, and it may be of value to assess how amoxicillin and other beta-lactam antibiotics interact with the band 3 protein in erythrocyte membranes, as well as the multitude of transmembrane signaling cascades.

Amoxicillin-induced hemolytic anemia presents a unique challenge due to the potential involvement of both immune and non-immune-mediated mechanisms. The role of G6PI and G6PD polymorphisms in susceptibility warrants further investigation. Another drug, primaquine, is an essential antimalarial drug that introduces a complex relationship between its metabolites, oxidative stress, and band 3 dysfunction in the context of G6PD deficiency.

### 2.3. Primaquine

Primaquine is a potent antimalarial medication utilized to combat malaria caused mainly by Plasmodium vivax, but it has been used to treat ovale and falciparum subtypes. It is under the class of aminoquinoline antimicrobials and is commonly used in conjunction with chloroquine as prophylaxis. However, recent utilization of primaquine demonstrates its efficacy as a radical cure for vivax malaria. In a general sense, primaquine aims to eliminate mature plasmodium parasites in the liver; however, it has also been shown to kill dormant hypnozoites, blood schizonts, and gametocytes, making it a relatively powerful agent against malarial infections [24].

#### 2.3.1. Mechanism of Action

Primaquine's mechanism of action is poorly understood, but several leading theories exist on how primaquine engages in gametocytocidal activity against plasmodium subtypes. Studies have shown that primaquine's hydroxylated metabolites may be involved in generating ROS, which are then converted back into H_2_O_2_, leading to parasite death [25].

#### 2.3.2. Metabolism

The method of action of primaquine depends on the formation of hydroxylated metabolites created from NADPH cytochrome P450 oxidoreductase activity. However, a deaminated metabolite named carboxy-primaquine does not have any antimalarial activity but does reach peak physiological levels tenfold higher than primaquine within 3–12 h [25].

#### 2.3.3. Hemolytic Anemia in G6PD Deficiency

**Figure 3:**
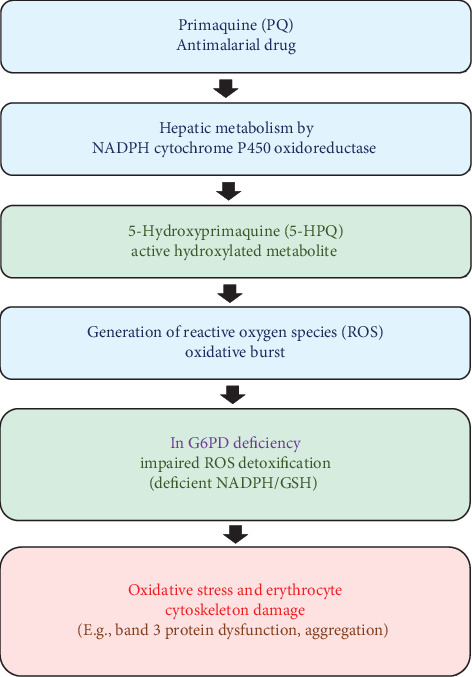
Primaquine metabolism and hemolytic pathway. This flow diagram illustrates the metabolism of primaquine into its active hydroxylated metabolite and its subsequent role in inducing oxidative stress and hemolytic anemia, particularly in individuals with glucose-6-phosphate dehydrogenase (G6PD) deficiency.

Primaquine has been shown to propagate hemolytic anemia in G6PD-deficient patients [26]. Metabolism of primaquine into 5-hydroxy primaquine (5-HPQ) has been associated with ROS generation, leading to oxidative burst of aged erythrocytes, not through classical lipid peroxidation, following a similar paradigm as dapsone metabolite-induced hemolytic anemia. It was shown that 5-HPQ oxidative injury was focused on erythrocyte cytoskeleton damage, though the precise mechanism of this damage remains unclear.  Figure 3 illustrates the flow diagram that depicts the metabolism of primaquine to 5-HPQ and its effects on RBCs, including oxidative stress.

#### 2.3.4. RBC Membrane Effects

A study by Bowman et al. [27] demonstrated that incubation of 5-HPQ with red erythrocytes resulted in rapid splenic sequestration upon damage, indicating that a signal was being propagated from membrane damage; however, the specific membranous target is not known. Theoretically, similar to the cellular response initiated by dapsone metabolites, it may be meritorious to investigate the role of regulatory phosphatases like SHP-2, which are likely recruited to counteract the band 3 hyperphosphorylation caused by toxic primaquine metabolites. It has already been shown that G6PD-deficient patients have weakened cytoskeletal integrity, potentially allowing for extraneous damage from toxic primaquine metabolites to ensue.

#### 2.3.5. Role of Band 3 and Plasmodium Interaction

A novel study done by Pantaleo et al. [28] demonstrates induction of band 3 dysfunction in plasmodium-affected erythrocytes via tyrosine phosphorylation, allowing for increased penetrance and release of merozoites. More importantly, phosphorylation of band 3 leads to membrane instability but not severe hemolytic anemia. Following this logic, it may be possible that primaquine's metabolite, 5-HPQ, in addition to existing membrane destabilization from both G6PD deficiency and plasmodium virulence, may allow for additional and irreparable erythrocyte membrane instability and subsequent splenic sequestration.

#### 2.3.6. Therapeutic Implications and Future Directions

The study by Pantaleo et al. [28] also showed the potential utilization of syk inhibitors as a protective agent against band 3 protein phosphorylation-induced membrane destabilization. It may be highly pertinent to assess the utilization of primaquine in conjunction with sky inhibitors to prevent resistance and achieve a desirable clinical outcome with a decreased incidence of hemolytic anemia in G6PD-deficient patients [29]. More studies on how the primaquine metabolite 5-HPQ interacts with band 3 and other transmembrane proteins are necessary to discover novel pharmacological methods to prevent erythrocyte membrane destabilization.

In summary, we have explored the intricate mechanisms underlying DIHA in G6PD deficiency, focusing on dapsone, amoxicillin, and primaquine. Dapsone metabolites, particularly DDS-NHOH, induce oxidative stress and band 3 dysfunction, leading to membrane instability. This is compounded by metabolic disruptions, including impaired glycolysis and PPP flux, as confirmed in recent *in vivo* models [7]. Amoxicillin may trigger hemolysis through both immune and non-immune pathways, with a potential role for G6PI and G6PD polymorphisms. Primaquine metabolites, notably 5-HPQ, exacerbate oxidative stress and contribute to band 3 dysfunction, further compromising RBC membrane integrity.

## 3. Conclusion

DIHA in individuals with G6PD deficiency remains a significant clinical challenge, particularly in regions where treatments for endemic diseases such as malaria and leprosy coincide with high prevalence of G6PD deficiency. This review highlights that the mechanisms driving hemolysis from drugs like dapsone, amoxicillin, and primaquine often involve complex pathways beyond traditional immune responses. These mechanisms center on drug metabolites, such as dapsone hydroxylamine and 5-hydroxyprimaquine, which generate ROS. The subsequent oxidative stress overwhelms deficient cellular defenses, leading to RBC membrane integrity damage. This process frequently implicates the crucial band 3 protein, which undergoes hyperphosphorylation. This damage signal triggers a compensatory response involving the recruitment of regulatory phosphatases like SHP-2. Recent in vivo evidence also indicates that DDS-NHOH-induced damage leads to dose-dependent clearance, with G6PD-deficient RBCs facing permanent removal due to metabolic lesions such as blocked glycolysis and pyruvate accumulation, suggesting a need to address both structural and metabolic therapeutic targets [7]. Based on these insights, future therapeutic development should explore promising interventions targeting the key drivers of this pathway. This includes developing Syk kinase inhibitors to block the initial pathological band 3 phosphorylation, or investigating strategies to enhance the function of protective phosphatases like SHP-2 to better counteract the oxidative damage. Additionally, modulating metabolic pathways, such as enhancing PPP flux or LDH activity, could mitigate the downstream effects of DDS-NHOH [7]. To effectively advance these solutions and improve patient outcomes, future research must prioritize specific, actionable goals such as elucidating the precise molecular interactions between drug metabolites and RBC membrane targets like band 3, further characterizing non-immune mediated hemolytic pathways, particularly for drugs like amoxicillin, analyzing how specific G6PD genetic variants modulate susceptibility to hemolysis from these key drugs, conducting preclinical evaluations of the potential targeted adjuvant therapies mentioned above (antioxidants, stabilizers, and kinase/docking inhibitors), and developing safer drug formulations or delivery systems for essential medicines that minimize toxic metabolite generation or oxidative stress. Pursuing these focused research directions is essential for creating improved pharmacological strategies and clinical protocols to protect the millions of G6PD-deficient individuals globally from DIHA.

## Figures and Tables

**Table 1:** Comparison of immune and non-immune mechanisms of DIHA in G6PD deficiency.

Feature	Immune mechanisms (DIIHA)	Non-immune mechanisms
Primary mediator	• Antibodies (drug-dependent or independent, e.g., IgG) and complement (e.g., C3 binding observed in an amoxicillin case study)	• Specific drug metabolites (e.g., Dapsone hydroxylamine/DDS-NHOH from dapsone, 5-hydroxyprimaquine/5-HPQ from primaquine), reactive oxygen species (ROS) generated by these metabolites
Underlying process	• Type II hypersensitivity reaction; immune response against drug, drug-RBC complex, or RBCs	• Direct chemical/oxidative damage to RBC components by drug/metabolites, especially pronounced in G6PD deficiency due to impaired ROS detoxification; potentially non-immune protein adsorption (NIPA) for some antibiotics (e.g., beta-lactamase inhibitors with amoxicillin)
Key molecular target(s)	• Drug adhered to RBC surface (forming complex), potentially specific RBC antigens targeted by antibodies	• Band 3 protein (undergoes hyperphosphorylation and aggregation, leading to dysfunction; this triggers the recruitment of regulatory phosphatases like SHP-2 in a cellular response to the damage), hemoglobin (cross-linking/precipitation into Heinz bodies), and RBC cytoskeleton (structural damage)
Cellular outcome	• Antibody/complement binding leading to RBC destruction (intravascular hemolysis and sequestration) or removal by the immune system	• Metabolite-induced oxidative stress causes membrane instability/remodeling, protein aggregation (Heinz bodies), cytoskeletal damage, leading to RBC deformation, and premature removal via splenic sequestration (observed with DDS-NHOH and 5-HPQ exposure)
Diagnostic clues	• Positive direct antiglobulin test (DAT)/Coombs test (detecting IgG or C3), as in amoxicillin-clavulanate case, presence of specific drug-dependent/independent antibodies	• Negative DAT/Coombs test (observed in some amoxicillin cases, G6PI deficiency case), absence of anti-reticulocyte antibodies (noted in G6PI deficiency case)
Contested area/focus	• Traditionally accepted mechanism, but challenged by findings of hemolysis without immune markers (e.g., some amoxicillin cases)	• Emerging understanding focusing on precise molecular interactions (metabolite-band 3 and SHP-2), role of oxidative stress, and mechanisms independent of antibody mediation, particularly for Dapsone and Primaquine in G6PD deficiency

## Data Availability

No underlying data were collected or produced in this study.
